# Cytokine Response Following SARS-CoV-2 Antigen Stimulation in Patients with Predominantly Antibody Deficiencies

**DOI:** 10.3390/v15051146

**Published:** 2023-05-10

**Authors:** Zane Lucane, Baiba Slisere, Gita Gersone, Sindija Papirte, Linda Gailite, Peteris Tretjakovs, Natalja Kurjane

**Affiliations:** 1 Department of Biology and Microbiology, Riga Stradins University, LV-1007 Riga, Latvia; zane.lucane@rsu.lv; 2 The Joint Laboratory, Pauls Stradins Clinical University Hospital, LV-1002 Riga, Latvia; 3 Department of Internal Diseases, Riga Stradins University, LV-1007 Riga, Latvia; 4 Department of Human Physiology and Biochemistry, Riga Stradins University, LV-1007 Riga, Latvia; 5 Faculty of Medicine, Riga Stradins University, LV-1007 Riga, Latvia; 6 Scientific Laboratory of Molecular Genetics, Riga Stradins University, LV-1007 Riga, Latvia; 7 Outpatient Clinic, Pauls Stradins Clinical University Hospital, LV-1002 Riga, Latvia; 8 Outpatient Clinic, Children’s Clinical University Hospital, LV-1004 Riga, Latvia

**Keywords:** antibody deficiency, common variable immunodeficiency, selective IgA deficiency, SARS-CoV-2, COVID-19, cytokine

## Abstract

Predominantly antibody deficiencies (PADs) are inborn disorders characterized by immune dysregulation and increased susceptibility to infections. Response to vaccination, including severe acute respiratory syndrome coronavirus 2 (SARS-CoV-2), may be impaired in these patients, and studies on responsiveness correlates, including cytokine signatures to antigen stimulation, are sparse. In this study, we aimed to describe the spike-specific cytokine response following whole-blood stimulation with SARS-CoV-2 spike peptides in patients with PAD (*n* = 16 with common variable immunodeficiency and *n* = 15 with selective IgA deficiency) and its relationship with the occurrence of coronavirus disease 2019 (COVID-19) during up to 10-month follow-up period. Spike-induced antibody and cytokine production was measured using ELISA (anti-spike IgG, IFN-γ) and xMAP technology (interleukin-1β (IL-1β), IL-4, IL-6, IL-10, IL-15, IL-17A, IL-21, TNF-α, TGF-β1). No difference was found in the production of cytokines between patients with PAD and controls. Anti-spike IgG and cytokine levels did not predict contraction of COVID-19. The only cytokine that distinguished between vaccinated and naturally infected unvaccinated PAD patients was IFN-γ (median 0.64 (IQR = 1.08) in vaccinated vs. 0.10 (IQR = 0.28) in unvaccinated). This study describes the spike-specific cytokine response to SARS-CoV-2 antigens, which is not predictive of contracting COVID-19 during the follow-up.

## 1. Introduction

Predominantly antibody deficiencies (PADs) are a group of disorders characterized by impaired B cell differentiation and decreased synthesis of immunoglobulins, resulting in recurrent sinopulmonary infections and, in some cases, poor vaccine response [1]. The most common diagnoses include selective IgA deficiency (SIgAD) and common variable immunodeficiency (CVID). Patients with SIgAD have reduced serum immunoglobulin A (IgA) levels below 0.07 g/L, with normal levels of other immunoglobulins. They are often asymptomatic and do not usually have an impaired response to vaccination. Symptomatic patients may experience recurrent respiratory and gastrointestinal infections and have a higher risk of atopic or autoimmune diseases compared to the general population [2]. CVID is the most common symptomatic antibody deficiency. The European Society for Immunodeficiencies Registry working definition for CVID is decreased levels of immunoglobulins (IgG, IgA, and/or IgM) and low switched memory B cells or poor vaccine response in individuals older than 4 years of age with no other identifiable cause or severe T cell defects [3]. Recurrent bacterial infections are the hallmark of the disease; however, the majority of patients also experience immune dysregulation-related non-infectious complications, including autoimmune diseases and malignancy [4]. Immune dysregulation, including dysregulated cytokine response, has been long known to be a part of the immunopathogenesis of predominantly antibody deficiencies, such as CVID and SIgAD [4,5,6,7].

When the severe acute respiratory syndrome coronavirus 2 (SARS-CoV-2) pandemic broke out in late 2019 [8], initial reports indicated increased disease-related morbidity and mortality in immunocompromised patients [9,10,11,12,13], including those with PAD [14,15,16]. After vaccines became available, several studies have investigated both the reactogenicity and immunogenicity of SARS-CoV-2 vaccines in PAD patients, and most studies concluded that the immune response in PAD patients was inferior to that in healthy vaccine recipients [17,18,19,20,21,22,23,24,25,26,27,28,29,30,31,32].

The main SARS-CoV-2 antigen is thought to be the spike (S) protein, which binds to the human angiotensin-converting enzyme 2 receptor via the receptor-binding domain of the virus and thus enters the cell. This protein consists of three parts: a signal peptide, an N-terminal S1 protease fragment containing the receptor-binding domain, and a C-terminal S2 protease fragment [33,34]. Coordinated cellular immunity is essential for disease control in viral infections, including SARS-CoV-2, while dysregulated and exacerbated inflammatory responses play a pivotal role in the development of severe coronavirus disease-19 (COVID-19) infection [35,36,37]. During natural infection with SARS-CoV-2, it has been observed that along with traditional markers of inflammation, such as C-reactive protein (CRP) and serum amyloid A, there are higher levels of specific pro-inflammatory cytokines, including interleukin 6 (IL-6), IL-1β, IL-8, IL-10, interferon gamma (IFN-γ), and tumor necrosis factor alpha (TNF-α) [38]. In addition, several other cytokines and chemokines have been shown to have altered expression in COVID-19, and the levels of some of these cytokines have been linked to the prognosis of COVID-19 [39,40,41,42,43,44,45,46,47,48,49,50,51,52,53,54,55,56,57,58,59]. However, some have speculated that in patients with inborn errors of immunity, immunodeficiency might act as a protective factor against the cytokine storm, which is the main trigger for the severe course of COVID-19 [60]. Therefore, the interplay between cytokine dysregulation in patients with PAD is intriguing; on the one hand, these patients may fail to mount an efficient immune response to vaccination, which could lead to a more severe course of the disease [61]. In contrast, a reduced inflammatory response could be related to a decreased risk of cytokine storms that lead to multi-organ failure [60].

In addition, attempts have been made to identify biomarkers related to efficient vaccine responses, and several associations between cytokine levels and SARS-CoV-2-specific humoral immune responses have been observed. Several studies with immunocompetent individuals have examined the correlates of SARS-CoV-2-specific humoral response and found changes in levels of M-CSF, IL-1α, IFN-γ, IL-1β, IL-10, IL-12p70, IL-6, IL-17A, IL-15, and IFN signaling-related cytokines (CXCL10, MCP-1, MCP-2, and MCP-3) to be correlated with SARS-CoV-2-specific antibody response in healthy volunteers [45,62,63,64,65]. Regarding PAD patients, several studies have examined the T cell response role in protection against SARS-CoV-2, showing IL-2 and/or IFN-γ secretion in response to pooled SARS-CoV-2 antigens [18,24,25,26,27,28]. Nonetheless, the understanding of the wider range of cytokines produced following SARS-CoV-2 antigen stimulation in individuals with predominantly antibody deficiencies is currently limited.

In this study, we aimed to describe the cytokine signatures following whole-blood stimulation with SARS-CoV-2 antigen in SARS-CoV-2 naturally infected and vaccinated PAD patients and compare these results with those of healthy controls. We also assessed whether cytokine profile or other immunological parameters were related to vaccine effectiveness for preventing coronavirus disease 2019 (COVID-19) during the follow-up period and cytokine signature relatedness with patients’ clinical parameters and anti-spike antibody levels.

## 2. Materials and Methods

### 2.1. Study Population

Patients with CVID and symptomatic SIgAD who were treated at a tertiary immunology center (Pauls Stradins Clinical University Hospital, Riga, Latvia) were enrolled in the study from April to July 2022. In total, 38 adult individuals were included in the study: 31 predominantly antibody deficiency patients (16 with CVID and 15 with SIgAD) and 7 healthy controls. All 31 patients were diagnosed according to the European Society for Immunodeficiencies diagnostic criteria [3]. To gather data on the demographic and clinical characteristics of patients, a thorough medical history of the patients was compiled, and their medical records were assessed. At the onset of the study, patient medical history was collected for several clinical parameters, including the frequency and type of infections (including SARS-CoV-2 infection and SARS-CoV-2 vaccination status), autoimmune diseases, levels of autoantibodies (rheumatoid factor, anti-nuclear antibody (ANA) and extractable nuclear antigen antibody (ENA) screening, antineutrophil cytoplasmic antibodies (p-ANCA, c-ANCA, atypical ANCA), antibodies against cardiolipins and phospholipids, lupus anticoagulant, anti-double-stranded DNA, anti-histone, anti-thyroid peroxidase, anti-tissue transglutaminase, anti-gliadin, and anti-gastric parietal cell antibodies), benign polyclonal lymphoproliferation (lymphadenopathy, splenomegaly, and hepatomegaly), granulomatous disease, enteropathy, atopy, or allergy (asthma, allergic rhinitis, food allergy, and atopic dermatitis), and malignant diseases. Data regarding patient immunoglobulin levels (IgG, IgM, and IgA) and lymphocyte subpopulations on the day of blood collection were also retrieved from the patients’ medical records. Lymphocytes were divided into the following subpopulations: B cells (CD19+), T cells (CD3+), T helper cells (CD3+CD4+), T cytotoxic cells (CD3+CD8+), T natural killer cells (CD3+CD56+), and activated T cells (CD3+HLADR+). The SARS-CoV-2 vaccination course was considered to be completed if the individual had received two doses if naïve to SARS-CoV-2 infection, or one dose if previously infected. The severity of COVID-19 in personal history was assessed using the World Health Organization Clinical Progress scale [66]. Immunological phenotyping was based on the EUROclass classifications [67]. CVID severity was assessed using the CVID severity score proposed by Ameratunga [68]. Following written consent to participate in this study, blood samples were obtained from patients and control participants. Four to ten months (122–317 days) after blood sample collection, the patient’s medical documentation (SARS-CoV-2 PCR test results, booster vaccination status) was reviewed again, and participants were contacted to determine if they had a positive SARS-CoV-2 rapid antigen test result during this time. In cases where a patient received an additional dose of the vaccine as a booster during the follow-up period, the duration of their follow-up time was modified to account for the period leading up to the date on which they received the booster dose. This study was conducted in accordance with the principles of the Declaration of Helsinki. The study protocol was reviewed and approved by the Central Board of the Ethics Committee of the Health Ministry of the Republic of Latvia (No. 01-29.1/2878).

### 2.2. Blood Collection

Peripheral venous blood samples were collected from all the participants. A peripheral blood sample for SARS-CoV-2 anti-spike IgG was collected in a serum collection tube, centrifuged after 30 min, and frozen at −20 °C until the day of measurement. A sample for peripheral blood mononuclear cell (PBMC) isolation and phenotyping of B and T lymphocyte subsets was collected in a lithium heparin-coated tube, and PBMCs were isolated as described previously [25]. Samples for the assessment of CD4+ and CD8+ cell cytokine responses to SARS-CoV-2 peptide stimulation and cytokine determination before/after SARS-CoV-2 peptide stimulation were collected in heparinized whole-blood QuantiFERON SARS-CoV-2 blood collection tubes (Qiagen, Hilden, Germany), incubated at 37 °C for 20 h, centrifuged according to the manufacturer’s protocol, and frozen at −20 °C for subsequent analysis. QuantiFERON SARS-CoV-2 blood collection starter kit contains four types of blood collection tubes: negative control, Ag1 tube (contains epitopes from the S1 fragment of the SARS-CoV-2 spike protein, measures response mediated by CD4+ cell response), Ag2 tube (contains epitopes from the S1 and S2 fragments of the SARS-CoV-2 spike protein, measures response mediated by both CD4+ and CD8+ cells), and positive control (mitogen control) [34,69]. Negative control was used to measure cytokine baseline levels and non-specific cytokine release during the incubation, while the Ag2 tube was used to measure cytokine levels after antigen stimulation.

### 2.3. Humoral Response to SARS-CoV-2

The humoral response to SARS-CoV-2 (IgG class antibodies to SARS-CoV-2 S1 domain of the spike protein) was assessed using the semi-quantitative enzyme-linked immunosorbent assay (ELISA) from Euroimmun (Anti-SARS-CoV-2 IgG assay, Euroimmun, Lübeck, Germany), following the manufacturer’s recommendations and as described previously. An IgGAM ratio (optical density compared with the calibrator) > 1.1 was considered a positive response. SARS-CoV-2 anti-spike response was considered low if the levels were <1.1, moderate if the anti-spike IgG levels were between 1.1 and 10, and high if levels were >10.

### 2.4. Cytokine Detection

Cytokine levels before and after SARS-CoV-2 S1 and S2 pool peptide stimulation were determined by Luminex xMAP technology using Luminex 200 equipment (A DiaSorin Company, Austin, TX, USA) and a multiplex assay (MILLIPLEX MAP Human TH17 Magnetic Bead Panel, Cat#: HTH17MAG-14 K and MILLIPLEX MAP TGF-ß1 Magnetic Bead Single Plex Kit, Cat#: TGFBMAG-64K-01, both from Merck Millipore, Darmstadt, Germany), following the manufacturer’s protocol. Levels of the following cytokines were measured: transforming growth factor beta 1 (TGF-ß1), IL-1β, IL-4, IL-6, IL-10, IL-15, IL-17A, IL-21, and TNF-α. Antigen-specific responses were quantified as the increase or decrease in cytokine levels in SARS-CoV-2 S1 and S2 pool peptide-stimulated samples compared with paired unstimulated negative controls. For the analysis, cytokines falling below the lowest detection limit were adjusted to 0.5, and absent values were removed.

Levels of interferon gamma (IFN-γ) production before and after SARS-CoV-2 S1 and S2 pool peptide stimulation were assessed using QuantiFERON SARS-CoV-2 ELISA assay (Qiagen, Hilden, Germany), as previously reported [69], according to the manufacturer’s protocol.

### 2.5. T and B Cell Subset Phenotyping

T and B cell subpopulations from freshly isolated PBMCs were determined by flow cytometry as described previously [25]. B cells were subdivided into the following subpopulations: naïve B cells (CD19+CD27−IgM+IgD+), marginal zone-like B cells (CD19+CD27+IgM++IgD+), switched memory B cells (CD19+CD27+IgM−IgD−), IgM-only memory B cells (CD19+CD27+IgM++IgD−), transitional B cells (CD19+IgD+CD27-IgM++CD38++), CD21low B cell (CD19+ IgM+,CD21-CD38-), plasmablasts (CD19+CD21+CD38+++IgM−), and atypical memory B cells (CD19+CD21−CD27−IgD−). T cells were subdivided as follows: naïve T helper cells (CD3+CD4+CD27+CD45RA+), central/transitory memory T helper cells (CD3+CD4+CD27+CD45RA−, effector memory T helper cells (CD3+CD4+CD27−CD45RA−), terminally differentiated T helper cells (CD3+CD4+CD27−CD45RA+), recent thymic emigrant T cells (CD3+CD4+CD31+CD45RO−), naïve T cytotoxic cells (CD3+CD8+CD27+CD45RA+), central/transitory memory T cytotoxic cells (CD3+CD8+CD27+CD45RA−), effector memory T cytotoxic cells (CD3+CD8+CD27−CD45RA−), terminally differentiated T cytotoxic cells (CD3+CD8+CD27−CD45RA+), T regulatory cells (CD3+CD4+CD25+FOXP3+CD127dim).

### 2.6. Statistical Analysis

The Shapiro–Wilk test was used to determine whether continuous variables were normally distributed. The results indicated that the data were not normally distributed; therefore, medians and interquartile ranges (IQRs) were used in data presentation, and nonparametric statistical methods were used in subsequent analysis. The differences in categorical variables were examined using the chi-square and Fisher exact tests. The Mann–Whitney U or Kruskal–Wallis test was used to compare continuous variables between two or more groups, respectively. The Wilcoxon signed-rank test was used to compare two dependent samples. Spearman’s rank test was used to assess the correlation between continuous variables. A binominal regression analysis was used to predict the occurrence of COVID-19. In binominal regression analysis, the dependent variable was whether COVID-19 was present during the follow-up period, while the independent variables were measured at the beginning of this period as follows: levels of anti-spike IgG, changes in cytokine levels (IFN-γ, TGF-β, IL-1β, IL-4, IL-6, IL-10, IL-15, IL-17A, IL-21, TNF-α) following whole-blood stimulation with SARS-CoV-2 S1 and S2 pool antigens, as well as levels of total IgG, IgA, and IgM, and different lymphocyte subpopulations as previously noted. Statistical significance was set at *p* value < 0.05. Statistical analysis was performed using IBM SPSS Statistics version 23 (IBM, New York, NY, USA). Graphs were generated using GraphPad Prism version 8 (GraphPad Software, San Diego, CA, USA).

## 3. Results

### 3.1. Study Population

Overall, 38 individuals were enrolled in the study: 31 patients with predominantly antibody deficiency (29.0% male, median age 40 years, IQR = 22) and seven control subjects (28.5% male, median age 50 years, IQR = 21). In the patient group, 16 and 15 patients were diagnosed with CVID and SIgAD, respectively. The clinical characteristics of the participants are presented in Table 1.

### 3.2. Prior Exposure to SARS-CoV-2 Antigen and COVID-19 during the Follow-Up Period in the Study Population

All patients and control group subjects had been previously exposed to the SARS-CoV-2 antigen at the time of blood sample collection via natural infection (4 patients, 0 controls), vaccination (12 patients, 3 controls), or both (15 patients, 4 controls). Of all vaccinated individuals (27 patients and 7 controls), the median time to completion of the vaccination regimen was 164 (IQR = 114) days: median 153 (IQR = 90) days in patients and 203 (IQR = 169) days in the healthy vaccine recipient group. The median duration between positive SARS-CoV-2 PCR test results and the date of evaluation for unvaccinated patients was 106 days (IQR = 144). Seven patients and two controls developed COVID-19 during the follow-up period, all of whom had mild disease according to the WHO classification (up to a score of 3) and were treated in an out-patient setting.

### 3.3. Changes in Cytokine Production Following SARS-CoV-2 Antigen Stimulation

To examine the changes in cytokine levels in response to SARS-CoV-2 antigen stimulation, we initially assessed the overall differences in cytokine production by comparing the baseline levels with those after stimulation in all study participants. Subsequently, we performed a similar analysis after excluding individuals who had not been vaccinated. Furthermore, we compared cytokine levels between the patient and control groups to determine whether significant differences existed in both baseline levels and changes in cytokine levels following antigen stimulation. Additionally, we examined the potential differences in cytokine levels and changes between patients diagnosed with common variable immunodeficiency (CVID) and selective IgA deficiency (SIgAD). All relevant statistical data are presented in Supplementary Table S1, which provides a detailed display of all pertinent statistical analyses.

**Figure 1:**
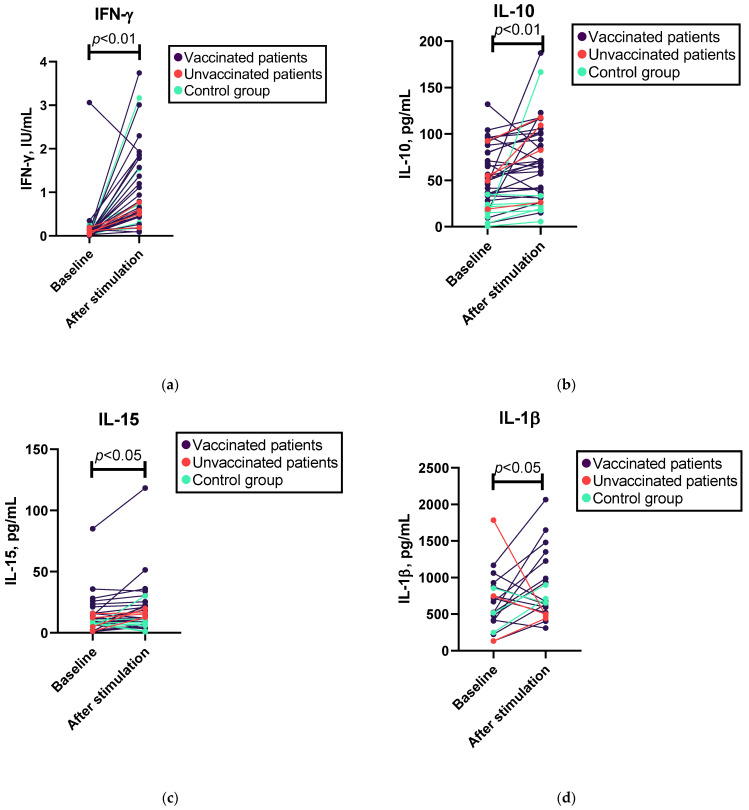

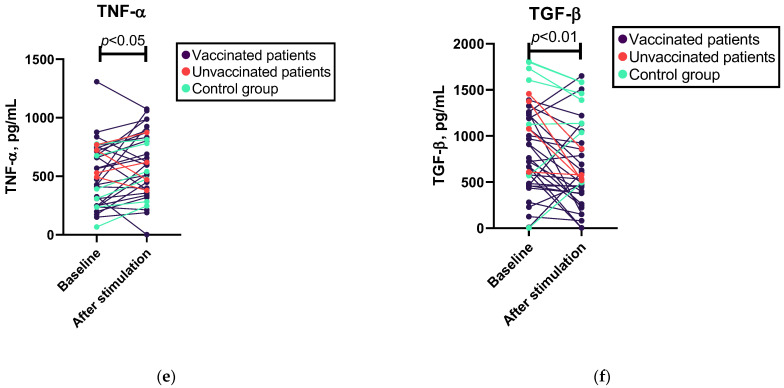
Wilcoxon test results for changes in cytokine levels following whole-blood SARS-CoV-2 antigen stimulation in patients and controls. (**a**) Levels of IFN-γ before and after SARS-CoV-2 antigen stimulation; *n* = 38. (**b**) Levels of IL-10 before and after SARS-CoV-2 antigen stimulation; *n* = 38. (**c**) Levels of IL-15 before and after SARS-CoV-2 antigen stimulation; *n* = 34. (**d**) Levels of IL-β before and after SARS-CoV-2 antigen stimulation; *n* = 20. (**e**) Levels of TNF-α before and after SARS-CoV-2 antigen stimulation; *n* = 36. (**f**) Levels of TGF-β1 before and after SARS-CoV-2 antigen stimulation; *n* = 38.

Overall, we found a statistically significant increase in the levels of IFN-γ, IL-10, IL-15, IL-17A, IL-1β, and TNF-α, but not IL-21, IL-4, and IL-6, and a decrease in TGF-β1 following SARS-CoV-2 antigen stimulation (see Figure 1 and Supplementary Table S1a). After excluding patients who had not been vaccinated, changes were observed in the levels of the cytokines mentioned previously, except for changes in IL-15, which were not statistically significant (see Supplementary Table S1a). However, in the Mann–Whitney U test, we did not observe a significant difference between the patient and control groups regarding the increase in cytokine levels after SARS-CoV-2 antigen stimulation (see Supplementary Table S1b). In contrast, such differences were observed in the baseline cytokine levels; patients were characterized by significantly elevated baseline levels of IL-10 and IL-4 compared with the control group (see Supplementary Table S1b).

In addition, when categorizing patients based on diagnosis, we observed significant changes in IFN-γ, IL-10, IL-15, and IL-4 levels in the SIgAD subgroup, whereas only changes in the levels of IFN-γ and TGF-β1 were observed in the CVID subgroup (see Supplementary Table S1a). We also found a statistically significant difference in the increase in IL-4 synthesis following SARS-CoV-2 antigen stimulation between different diagnosis groups: IL-4 did not increase in the CVID group (median 0 ng/mL), but we found a median 1.2 ng/mL increase in the SIgAD group (see Supplementary Table S1b).

### 3.4. Predictors of COVID-19 during the Follow-Up Period in PAD Patients

To determine potential clinical parameters that may be associated with the occurrence of COVID-19 during the follow-up period, we conducted a binomial logistic regression analysis and employed the Mann–Whitney U test. All the relevant statistical data are presented in Supplementary Table S2.

**Figure 2:**
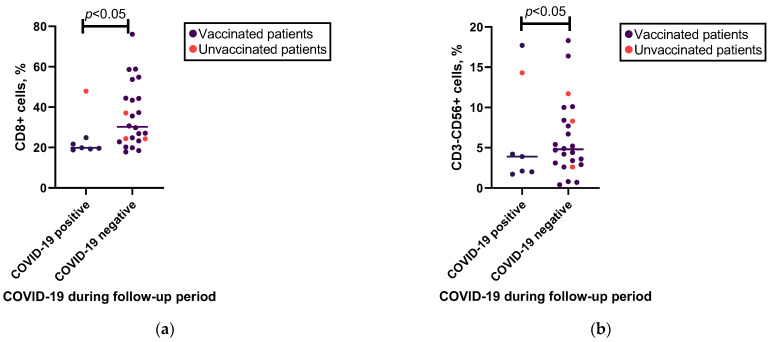
Associations of cytotoxic T cell and NK cell percentages with occurrence of COVID-19 in follow-up. (**a**) Mann–Whitney U test results for associations between cytotoxic T (CD8+) cells and occurrence of COVID-19 in follow-up in patients who did (*n* = 7) or did not (*n* = 24) have COVID-19 during the follow-up period. (**b**) Mann–Whitney U test results for associations between NK (CD3−CD56+) cells and occurrence of COVID-19 in follow-up in patients who did (*n* = 7) or did not (*n* = 24) have COVID-19 during the follow-up period.

Binomial logistic regression analysis indicated that variables such as anti-spike IgG levels, cytokine levels following SARS-CoV-2 antigen stimulation, total IgG, IgM, and IgA levels, and lymphocyte subpopulations cannot be used to predict the likelihood of contracting COVID-19 (see Supplementary Table S2a). However, the Mann–Whitney U test demonstrated significant differences in cytotoxic T and NK cell subpopulations between patients who contracted COVID-19 during the follow-up period and those who did not (see Figure 2 and Supplementary Table S2b). However, the effect size of this test was moderate. After excluding patients who had not been vaccinated, significant differences in cytotoxic T cells, but not NK cells, were observed (see Supplementary Table S2b).

When categorizing patients based on their diagnosis, a consistent observation of differences in cytotoxic T cells, but not NK cells, was found in the CVID group, while in the SIgAD group, we did not find a statistically significant difference in lymphocyte subpopulations between the patients who contracted COVID-19 and those who did not.

We did not find a statistically significant relationship between the occurrence of COVID-19 during the follow-up period and anti-spike IgG or cytokine levels after the SARS-CoV-2 antigen stimulation (see Supplementary Table S2b).

### 3.5. Associations between the Cytokine Levels and Previous SARS-CoV-2 Vaccination

To ascertain potential differences in cytokine levels between vaccinated and unvaccinated patients and controls, the Kruskal–Wallis test was conducted. Furthermore, we assessed whether the cytokine response was correlated with the type of vaccination received within the patient group. All the relevant statistical data are presented in Supplementary Tables S3 and S6.

**Figure 3:**
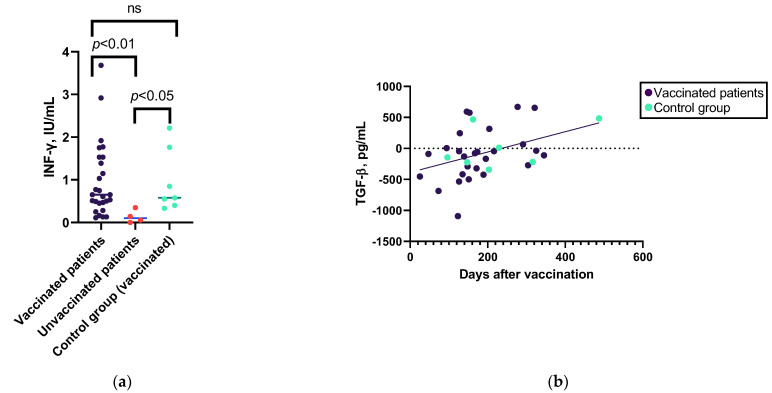
Associations between the cytokine levels and previous SARS-CoV-2 vaccination. (**a**) Mann–Whitney U test results for associations between the IFN-γ level changes after SARS-CoV-2 antigen stimulation in vaccinated patient group (*n* = 28), unvaccinated patient group (*n* = 4), and control group (*n* = 7). (**b**) Spearman’s correlation between the increase in levels of TGF-β1 after whole-blood stimulation with SARS-CoV-2 spike protein S1 and S2 pool peptides in vaccinated patients (*n* = 27) and control group individuals (*n* = 7). ns—non-significant.

The Kruskal–Wallis test indicated a significant difference in the median increase in IFN-γ levels in the vaccinated, unvaccinated, and control groups (see Figure 3a and Supplementary Table S3a). When categorizing patients based on their diagnosis, a consistent observation of a difference between vaccinated and unvaccinated individuals was found in the CVID group, but not in the SIgAD group (see Supplementary Table S3b). No other significant differences were found in the increase in cytokine levels after SARS-CoV-2 antigen stimulation between the vaccinated and unvaccinated patients or vaccinated controls.

Furthermore, there was no significant correlation between the type of vaccine administered and changes in cytokine levels (see Supplementary Table S3c).

Regarding the cytokine response correlation with time after the last vaccine dose, the only cytokine whose level changed after antigen stimulation correlated with time after vaccination was TGF-β1 (in the patient group) (Figure 3b and Supplementary Table S6c).

### 3.6. Associations between the Cytokine Levels and SARS-CoV-2 Humoral Response

We also assessed whether the cytokine response to SARS-CoV-2 antigen stimulation was related to the anti-spike IgG antibody levels. All the relevant statistical data are presented in Supplementary Tables S4 and S6.

**Figure 4:**
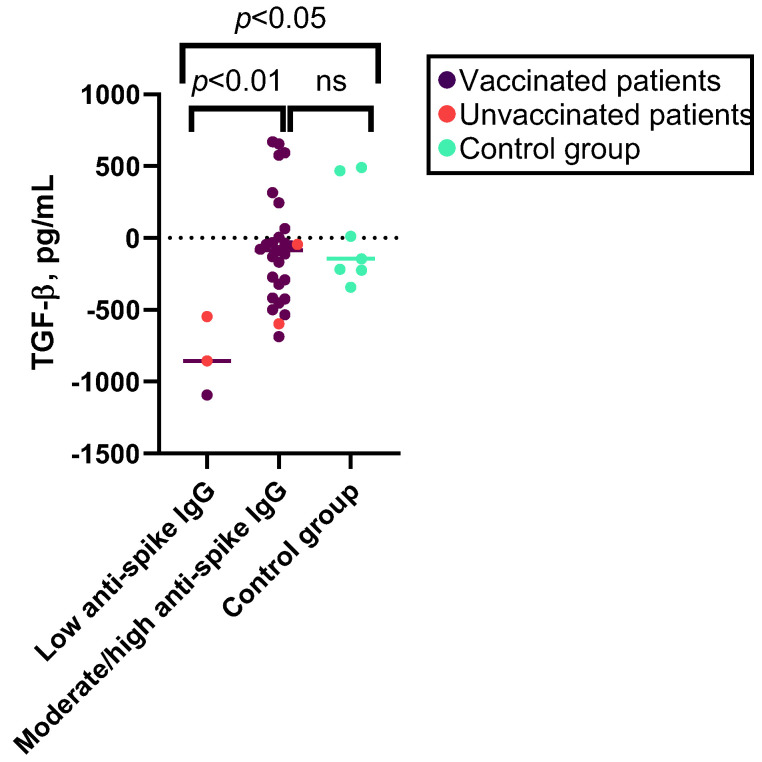
Kruskal–Wallis test results for associations between the levels of TGF-β1 after SARS-CoV-2 antigen stimulation and SARS-CoV-2 humoral response. Median decrease in levels of TGF-β1 after SARS-CoV-2 antigen stimulation in patients with low (*n* = 3), moderate, or high anti-spike IgG response (*n* = 28) and controls (*n* = 7). ns—non-significant.

No significant correlation was observed between the levels of anti-spike IgG and the increase in cytokine levels following SARS-CoV-2 antigen stimulation in patients with PAD (see Supplementary Table S6c). However, when categorizing patients based on low, moderate, or high anti-spike antibody response, patients with low antibody response exhibited a higher median decrease in TGF-β1 levels after stimulation with SARS-CoV-2 antigen and compared to patients with a moderate or high humoral response or control group individuals (see Figure 4 and Supplementary Table S4a).

### 3.7. Associations between the Cytokine Levels and Patient’s Demographic and Clinical Characteristics

We also assessed whether there were any correlations between age and cytokine responses to SARS-CoV-2 antigen stimulation as well as any differences in cytokine responses in relation to various clinical manifestations. All relevant statistical data are presented in Supplementary Tables S5 and S6.

In the patient group, age was significantly correlated with changes in the levels of TGF-β1 and IL-1β following SARS-CoV-2 antigen stimulation (see Supplementary Table S6a).

**Figure 5:**
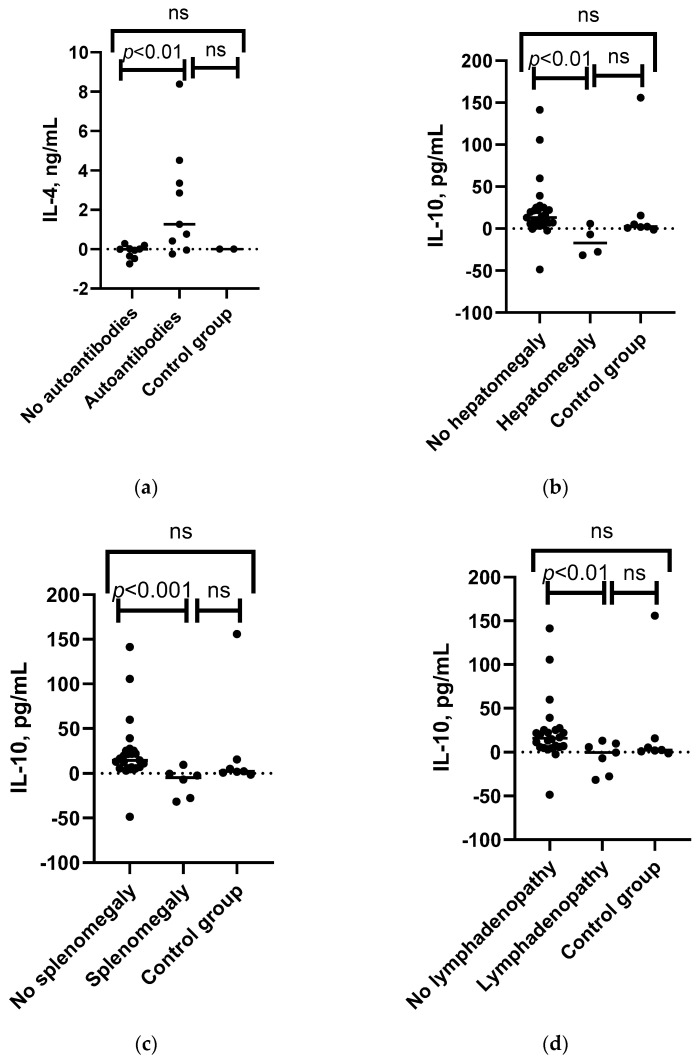

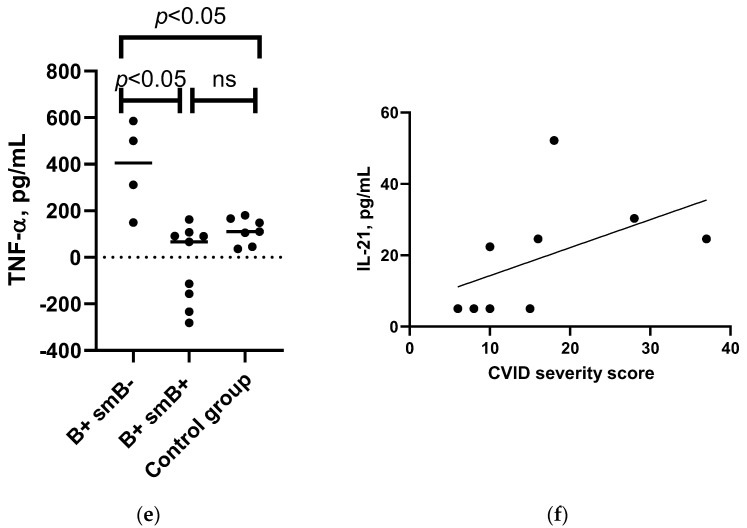
Associations between the IL-4, IL-10, TNF-α, and IL-21 levels and patient’s clinical characteristics. (**a**) Mann–Whitney U test results for associations between IL-4 level changes after SARS-CoV-2 antigen stimulation in patients with detected autoantibodies (*n* = 8), patients without the condition (*n* = 8), and control group (*n* = 2). (**b**) Mann–Whitney U test results for associations between IL-10 level changes following SARS-CoV-2 antigen stimulation in patients with hepatomegaly (*n* = 4), patients without the condition (*n* = 27), and control group (*n* = 7). (**c**) Mann–Whitney U test results for associations between IL-10 level changes following SARS-CoV-2 antigen stimulation in patients with splenomegaly (*n* = 5), patients without the condition (*n* = 26), and control group (*n* = 7). (**d**) Mann–Whitney U test results for associations between the changes in IL-10 level following SARS-CoV-2 antigen stimulation in patients with lymphadenopathy (*n* = 7), patients without the condition (*n* = 24), and control group (*n* = 7). (**e**) Mann–Whitney U test results for associations between the changes in TNF-α following SARS-CoV-2 antigen stimulation in patients with B+SmB- (*n* = 4), B+SmB+ phenotype (*n* = 9), and control group individuals (*n* = 7). (**f**) Spearman’s correlation between the CVID patients’ severity score and increase in levels of IL-21 after whole-blood stimulation with SARS-CoV-2 antigen in CVID patients (*n* = 9). ns—non-significant.

**Figure 6:**
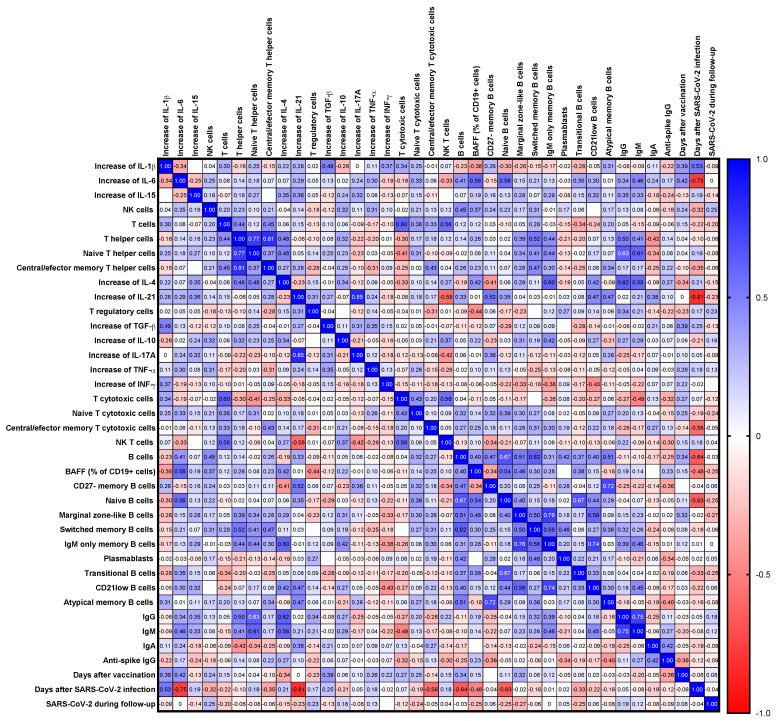
Correlation matrix of the changes in levels of cytokines following SARS-CoV-2 antigen stimulation and immunological parameters. Spearman r values are shown from red (−1.0) to blue (1.0). The absence of an r value in certain blank cells signifies that the corresponding Spearman’s correlation coefficient (r) value is 0.

Distinct variations were observed in the cytokine profiles when comparing various complications and comorbidities. The Kruskal–Wallis test indicated that there was a significant difference in the median increase in the level of IL-4 and the frequency of detected autoantibodies in personal medical history, while in the control group or patients without autoantibodies, no increase was observed; in patients who had detected autoantibodies in their personal medical history, we found a median 1.26 ng/mL increase in the levels of IL-4 following SARS-CoV-2 antigen stimulation (see Figure 5a and Supplementary Table S5a). Additionally, baseline IL-4 and TNF-α levels were higher in patients with autoantibodies. We found no association between increased cytokine levels, including IL-4, after SARS-CoV-2 antigen stimulation and clinically detectable autoimmune diseases (see Supplementary Table S5a). However, when categorizing patients according to diagnosis, in the CVID group, TGF-β1 levels were related to the presence of autoimmune disease (see Supplementary Table S5c). An increase in the level of IL-4 following SARS-CoV-2 antigen stimulation, but not the baseline IL-4 level, was also correlated with the levels of total IgG and total IgM (see Figure 6 and Supplementary Table S6c).

Changes in IL-10 levels after SARS-CoV-2 antigen stimulation were found to be significantly associated with lymphadenopathy, splenomegaly, and hepatomegaly. IL-10 levels decreased after SARS-CoV-2 antigen stimulation in patients with lymphadenopathy, whereas an increase was observed in those without the condition and in the control group. A similar trend was observed for splenomegaly and hepatomegaly, as well as for changes in IL-15 levels and splenomegaly (see Figure 5b–d and Supplementary Table S5a). When categorizing patients based on their diagnosis, a consistent observation of changes in IL-10 levels was found in the CVID group, but not in the SIgAD group, since only one patient with each of these conditions was present in the SIgAD subgroup (see Supplementary Table S5c,d).

In the CVID patient subgroup, we also assessed the association with the EUROclass classification groups and found a statistically significant difference between the increase in levels of TNF-α after SARS-CoV-2 stimulation; patients with low switched memory B cell percentages (EUROclass group B+SmB-) had a higher increase in TNF-α levels compared to patients with normal switched memory B cell percentages or the control group subjects (see Figure 5e and Supplementary Table S5b). There was only one patient with B- and one patient with CD21_low_ high phenotype and two with transitional cell high phenotype; therefore, the median values for these patients have not been reported.

In the CVID patient subgroup, we assessed a correlation with the CVID severity score, and only spike-induced IL-21 response correlated with the severity of CVID (rs = 0.809, *p* = 0.009, *n* = 9).

### 3.8. Correlation between the Cytokine Levels and Lymphocyte Subsets

Correlations between changes in cytokine levels following SARS-CoV-2 antigen stimulation and immunological parameters are shown in Figure 6 and Supplementary Table S6.

Overall, we found a significant correlation between the increase in the levels of Th17 cytokines (IL-17A and IL-21) after SARS-CoV-2 antigen stimulation, as well as TGF-β with the pro-inflammatory cytokines IL-1β and TNF-α (see Supplementary Table S6b). We also observed a correlation between the baseline levels of pro-inflammatory cytokines: IL-1β correlated with the levels of IL-6 and TNF-α, and IL-6 with TNF-α and IL-10 correlated with IL-4 and IL-15 (see Supplementary Table S6a).

Regarding correlations with lymphocyte subsets, several B cell subsets were correlated with changes in cytokine levels after SARS-CoV-2 antigen stimulation: marginal zone-like B cells and IgM-only memory B cells correlated with changes in IFN-γ and IL-10 levels, while IFN-γ was also correlated with CD21low B cells. Changes in IL-4 levels also correlated with IgM-only memory B cells, CD21low B cells, and T helper cells. Changes in IL-1β levels were related to total and atypical B cells, whereas changes in IL-6 levels were related to naïve B cells. Natural killer T cells correlated with changes in IL-10, IL-17A, and IL-21 levels. Changes in IL-17A levels also correlated with total T cells and terminally differentiated T cells. Natural killer cells were correlated with changes in IL-17A and TNF-α levels (see Figure 6 and Supplementary Table S6c).

## 4. Discussion

In this study, we present the spike-specific cytokine response observed after whole-blood stimulation with the SARS-CoV-2 antigen in naturally infected unvaccinated and vaccinated PAD patients and explore the cytokine profile and other immunological parameters as markers for predicting the contraction of COVID-19 infection during the up to 10-month follow-up period.

Overall, we were not able to predict the occurrence of COVID-19 based on the anti-spike humoral response, baseline cytokine levels, changes in cytokine levels following whole-blood SARS-CoV-2 antigen stimulation, or other immunological parameters in patients with PAD. However, patients with higher percentages of cytotoxic T and NK cells showed a lower incidence of COVID-19 during the follow-up period. This finding is consistent with the current understanding that these specific cell types play a crucial role in the antiviral immune response, including that against SARS-CoV-2 [70]. Functional exhaustion of cytotoxic lymphocytes (such as CD8+ cytotoxic T cells and NK cells) has been associated with poor COVID-19 prognosis [33,44], whereas substantial CD8+ T cell responses have been associated with mild COVID-19 disease [71]. Cytokine levels after SARS-CoV-2 antigen stimulation did not prove to be predictive of COVID-19. To the best of our knowledge, there have been no prior studies examining the potential of cytokine levels as predictive markers of occurrence of COVID-19 in patients with PAD; however, previous research conducted on healthy individuals during an 8-month follow-up period following CoronaVac vaccination found that those who exhibited lower levels of IFN-γ in the IFN-γ release assay were at a higher risk of contracting COVID-19 [72]. Similarly, we were unable to predict the likelihood of being infected with SARS-CoV-2 through anti-spike antibody levels, which is consistent with the findings of the COV-AD study [72].

Cytokines that have previously exhibited alterations in their levels following vaccination in immunocompetent individuals include IFN-γ, IL-10, IL-15, IL-17A, IL-1 β, TNF-α, IP-10/CXCL10, IL-6, IFN-α2, IL-12p70, IL-18, IL-23, and IL-33 [73,74,75,76,77,78]. Interestingly, in addition to several of the mentioned cytokines, we also observed a decrease in the level of TGF-β1 following whole-blood stimulation with the SARS-CoV-2 antigen, and this decrease was more prominent in patients who showed a low anti-spike IgG response as well as in CVID patients with an autoimmune disease. The main source of TGF-β1 is epithelial cells, but it can be produced by most immune cells in response to infection, and it mainly acts as a regulator of multiple types of immune cells, including T regulatory (Treg) cells, NK cells, and macrophages [79]. The TGF-β1 role in antibody synthesis is mainly induced by the induction of T regulatory cells to suppress B cells [80,81] and the induction of an isotype switch to mainly IgA1 and IgA2 class antibodies [82]. In a study on the dynamics of adaptive immune response in severe COVID-19 examining the plasmablast transcriptome changes over the course of eight weeks, plasmablasts showed a continuous immune reaction; during the first week, plasmablasts showed an immune response directed against SARS-CoV-2, characterized by the synthesis of IgG antibodies against the spike and nucleocapsid proteins, but later response switched to IgA-expressing plasmablasts, which were are not specific to SARS-CoV-2 proteins and reflected continued instruction of the B lymphocytes by TGF-β1 [82]. In addition, in the upper airways of immunocompetent patients, TGF-β1 transcript level expression was lower in SARS-CoV-2-infected patients than in controls, and in asymptomatic individuals, TGF-β1 correlated negatively with IFN-γ, suggesting its role in the regulation of early antiviral inflammatory response [83]. In our cohort, changes in the levels of TGF-β1 were significantly correlated with the levels of IL-1β and TNF-α. Indeed, TGF-β1 can activate nuclear factor-kappa-light-chain-enhancer of activated B cells (NF-kB), which can further upregulate various cytokines, including IFN-γ, TNF-α, and IL-1 β [84,85]. Further research is warranted to determine the function of TGF-β1 in the adaptive immune reactions related to COVID-19 in patients with PAD.

This report confirms a higher IFN-γ response to SARS-CoV-2 antigen stimulation in vaccinated PAD patients than in unvaccinated patients who were exposed to the SARS-CoV-2 virus via natural COVID-19, similar to that previously reported in individuals in the general population [74,86], although conflicting evidence exists [87]. In our cohort, we found no difference in the changes in other cytokine levels after SARS-CoV-2 antigen stimulation when comparing vaccinated and unvaccinated patients, although previously in a study with healthy individuals, TNF-α levels in response to stimulation with peptide pools corresponding to the SARS-CoV-2 spike, nucleocapsid, or membrane protein were significantly higher in individuals who had completed a vaccination regimen than in unvaccinated individuals [74]. These differences could be because we only examined cytokine responses to S1 and S2 pools in this study.

We also examined whether the spike-specific cytokine response was associated with any specific non-infectious complications. In this study, we found an association between the magnitude of the increase in IL-4 and the presence of autoantibodies in patients’ serum, as well as an increase in IL-4 correlated with total IgG and IgM levels, but not with anti-spike IgG. This finding is in line with a study of COVID-19 patients where excessive plasmablast expansion was correlated with autoantibody production, and these plasmablasts developed according to IL-4− and BAFF-driven developmental trajectories. Although they were not enriched in autoreactive B cells, two distinct memory populations (CD80+/ISG15+ and CD11c+/SOX5+/T-bet+/−) with signs of autoreactivity were identified, which were considered to be the source of COVID-19 autoantibodies [88]. Indeed, the evidence of the development of autoimmune conditions following COVID-19 has accumulated during the past few years [44,89,90,91,92,93,94,95]. In addition, we found a correlation between IL-21 and CVID severity score in patients with CVID. Within secondary lymphoid organs, T follicular helper (Tfh) cells are primarily engaged in ensuring B cell survival, proliferation, and differentiation by producing significant quantities of IL-21 and IL-4 [96], and the majority of inborn errors of immunity patients exhibit spike-specific circulating Tfh cells [28]. Regarding CVID and SIgAD patients, association studies suggest that defective IL-4 and IL-21 signaling has been linked to an increased prevalence of non-infectious complications, including autoimmunity [4,97,98,99,100].

IL-10 was identified as another cytokine whose increase was associated with a specific patient phenotype. IL-10 is an anti-inflammatory cytokine that is capable of inhibiting the synthesis of pro-inflammatory cytokines and is produced mainly by T cells, especially Treg and Tfh cells, as well as monocytes and B regulatory cells [7]. In our study, the extent of the spike-specific IL-10 response was significantly lower in patients with benign polyclonal lymphoproliferation, manifested as hepatomegaly, splenomegaly, and lymphadenopathy. In the EUROclass trial, splenomegaly was associated with dysregulation of B cell homeostasis, including reduced switched-low memory B cells, which could indicate dysregulated germinal center development [67]. We also observed that the level of the pro-inflammatory cytokine TNF-α was significantly higher in patients with low switched memory B cell counts (EUROclass B+SmB−). Although the cytokine response to SARS-CoV-2 antigen has not been examined previously in the context of non-infectious complications of PAD, the response of circulating Tfh isolated from CVID patients to submitogenic PHA+IL-2 stimulation, as indicated by the intracellular expression of Tfh cytokines (IL-4, IL-10, IL-21), with non-infectious complications (autoimmunity and/or granulomatous disease) was lower than that in patients without these complications. In contrast, the IL-4 response was higher in patients with autoimmunity and/or granulomatous disease [101]. Studies of IL-10 levels without antigen stimulation suggest that low IL-10 production in patients with CVID-like disorders with homozygous deletion of the inducible T cell co-stimulator (ICOS), a key receptor of Tfh help to germinal center B cells, may contribute to the disturbed germinal center reaction in secondary lymphoid organs, and patients with this genetic defect can be associated with splenomegaly [102]. In addition, CVID patients have been shown to have a decreased frequency of naïve regulatory T cells, one of the major sources of IL-10, in CVID patients with splenomegaly [103].

Several major limitations should be considered. First, the sample size was limited due to the rarity of these conditions, which reduced the statistical power and limited the ability to detect significant associations; therefore, the utility of predicting factors should be studied in larger cohorts. Second, the timeframe within the measurements after completion of the immunization schedule was broad in this cohort. Third, whole-blood stimulation has limitations that should be considered: T cell responses are complex, and natural or vaccine-induced T cell stimulation can activate different cell subpopulations and molecular pathways, resulting in distinct functional cytokine expression profiles and clinical outcomes. In addition, we examined only the spike-induced cytokine response, and other SARS-CoV-2 proteins, such as the nucleocapsid or membrane proteins, were not included. The cytokine IL-2 was not included in the analysis due to technical reasons. Therefore, it is possible that predicting vaccine-induced or natural protection may be more accurate if we consider the activation of T cells that express different cytokines or activation markers in addition to those currently being examined. In addition, there is a limitation in comparing our results with those of other studies, because the methods of investigation vary significantly between studies.

## 5. Conclusions

No significant association was observed between spike-specific cytokine response, anti-spike IgG levels, or other relevant factors and the likelihood of contracting COVID-19. Similar to the healthy control groups, the most distinct difference between vaccinated and naturally infected unvaccinated patients with PAD was the higher increase in IFN-γ levels in the vaccinated patients.

## Figures and Tables

**Table 1:** Demographic and clinical parameters of the predominantly antibody deficiency patients.

Diagnosis	Sex	Age	Vaccination to SARS-CoV-2	Days after Vaccination	COVID-19 in Personal Medical History Prior to Blood Collection	COVID-19 during Follow-Up Period	Clinical Manifestations and Non-Infectious Complications	Infection-Related Complications
CVID	Female	30	Pfizer BioNTechBNT162b2	153	Yes, 118 days after COVID-19, WHO score * 2	No	Recurrent pneumonia, otitis media, tonsillitis	Bronchiectasis
CVID	Female	50	Spikevax mRNA-1273	139	No	No	Recurrent pneumonia, otitis media, skin infections; viral warts; allergic rhinitis; lymphadenopathy, splenomegaly	Bronchiectasis
CVID	Female	27	Spikevax mRNA-1273	126	Yes, 55 days after COVID-19, WHO score 2	No	Recurrent pneumonia, sinusitis, osteomyelitis; allergic rhinitis; celiac disease; Hashimoto thyroiditis; type I diabetes mellitus	No
CVID	Female	28	Pfizer BioNTechBNT162b2	151	No	No	Recurrent pneumonia, sinusitis, tonsillitis, skin infections; atopic dermatitis; psoriasis; lymphadenopathy, hepatosplenomegaly	No
CVID	Female	35	Jcovden Ad26.CoV2	146	Yes, 387 days after COVID-19, WHO score 2	No	Recurrent pneumonia, sinusitis, urinary tract infections; viral warts; atopic dermatitis; Hashimoto thyroiditis; lymphadenopathy	Bronchiectasis, hearing impairment due to recurrent otitis media
CVID	Male	40	Pfizer BioNTechBNT162b2	128	No	No	Recurrent pneumonia, sinusitis, meningitis; atopic dermatitis; splenomegaly	No
CVID	Male	37	Spikevax mRNA-1273	345	Yes, 100 days after COVID-19, WHO score 2	No	Recurrent pneumonia, otitis media, sinusitis	Bronchiectasis
CVID	Male	28	Pfizer BioNTechBNT162b2	173	No	Yes	Recurrent pneumonia, otitis media, sinusitis, skin infections; severe EBV infection in anamnesis; lymphadenopathy	No
CVID	Female	58	Spikevax mRNA-1273	123	Yes, 41 days after COVID-19, WHO score 2	No	Recurrent pneumonia, otitis media; asthma; primary biliary cholangitis; lymphadenopathy, hepatosplenomegaly; non-infectious diarrhea	Hearing impairment due to recurrent otitis media
CVID	Female	61	Pfizer BioNTechBNT162b2	171	Yes, 453 days after COVID-19, WHO score 6	No	Recurrent pneumonia, otitis media, tonsilitis; asthma; lymphadenopathy, hepatosplenomegaly	Bronchiectasis
CVID	Female	55	Pfizer BioNTechBNT162b2	167	No	Yes	Recurrent pneumonia, sinusitis, tonsillitis; allergic rhinitis; non-infectious diarrhea; meningioma	No
CVID	Male	37	Pfizer BioNTechBNT162b2	321	Yes, 86 days after COVID-19, WHO score 5	No	Recurrent pneumonia; non-infectious diarrhea	Bronchiectasis
CVID	Female	63	Spikevax mRNA-1273	189	No	No	Recurrent pneumonia, otitis media, sinusitis, tonsillitis, skin infections, meningitis	Bronchiectasis, hearing impairment due to recurrent otitis media
CVID	Male	69	Pfizer BioNTechBNT162b2	216	Yes, 98 days after COVID-19, WHO score 5	Yes	Recurrent pneumonia, tonsillitis; asthma; chronic lymphocytic leukemia of B cell type	Bronchiectasis
CVID	Female	48	No vaccination	N/A	Yes, 73 days after COVID-19, WHO score 4	No	Recurrent pneumonia; enteropathy	No
CVID	Male	45	No vaccination	N/A	Yes, 358 days after COVID-19, WHO score 1	No	Recurrent pneumonia, otitis media, sinusitis, osteomyelitis, recurrent *Herpes Zoster*; vitiligo	Bronchiectasis
SIgAD	Female	37	Spikevax mRNA-1273	325	Yes, 95 days after COVID-19, WHO score 2	No	Angioedema; Hashimoto thyroiditis	No
SIgAD	Female	19	Pfizer BioNTechBNT162b2	277	No	No	Recurrent pneumonia, otitis media, urinary tract infections, tonsillitis, sepsis in anamnesis; allergic rhinitis; type 1 diabetes	No
SIgAD	Female	53	Pfizer BioNTechBNT162b2	94	Yes, 526 days after COVID-19, WHO score 4	No	Recurrent skin infections; rheumatoid arthritis, drug-induced osteoporosis with pathological fractures	No
SIgAD	Female	30	Pfizer BioNTechBNT162b2	47	No	No	Recurrent sinusitis, tonsillitis; atopic dermatitis asthma; lymphadenopathy, splenomegaly; non-infectious diarrhea	No
SIgAD	Female	51	No vaccination	N/A	Yes, 105 days after COVID-19, WHO score 2	No	Lichen ruber planus in oral cavity mucosa	No
SIgAD	Male	21	Pfizer BioNTechBNT162b2	195	No	No	Atopic dermatitis; psoriasis; non-infectious diarrhea	No
SIgAD	Female	52	Pfizer BioNTechBNT162b2	135	No	No	Recurrent tonsillitis; allergic rhinitis; idiopathic urticaria	No
SIgAD	Female	68	Spikevax mRNA-1273	73	No	Yes	Recurrent urinary tract infections; rheumatoid arthritis; gastric cancer in anamnesis	No
SIgAD	Female	25	Pfizer BioNTechBNT162b2	291	Yes, 345 days after COVID-19, WHO score 2	No	Recurrent urinary tract infections, tonsillitis; viral warts; atopic dermatitis; non-infectious diarrhea	No
SIgAD	Male	44	Spikevax mRNA-1273	304	Yes, 77 days after COVID-19, WHO score 2	No	Recurrent skin infections; atopic dermatitis, eosinophilic esophagitis; celiac disease, Hashimoto thyroiditis	No
SIgAD	Female	42	Pfizer BioNTechBNT162b2	126	Yes, 59 days after COVID-19, WHO score 2	No	Recurrent tonsillitis	No
SIgAD	Female	34	Spikevax mRNA-1273	148	Yes, 35 days after COVID-19, WHO score 2	No	Recurrent tonsillitis, meningitis in anamnesis, recurrent *Herpes Zoster*; allergic rhinitis	No
SIgAD	Female	36	No vaccination	N/A	Yes, 108 days after COVID-19, WHO score 2	Yes	Recurrent otitis media, sinusitis	No
SIgAD	Female	41	Pfizer BioNTechBNT162b2	25	Yes, 244 days after COVID-19, WHO score 2	Yes	Recurrent tonsillitis; hepatomegaly	No
SIgAD	Male	32	Pfizer BioNTechBNT162b2	204	No	Yes	Recurrent sinusitis, tonsillitis; atopic dermatitis	No
Control	Female	52	Pfizer BioNTechBNT162b2	487	No	No	N/A	N/A
Control	Male	37	Pfizer BioNTechBNT162b2	316	Yes, 140 days after COVID-19, WHO score 2	No	N/A	N/A
Control	Male	50	Spikevax mRNA-1273	147	Yes, 140 days after COVID-19, WHO score 2	No	N/A	N/A
Control	Female	50	Pfizer BioNTechBNT162b2	96	No	No	N/A	N/A
Control	Female	31	Spikevax mRNA-1273	162	Yes, 774 days after COVID-19, WHO score 2	No	N/A	N/A
Control	Female	30	Pfizer BioNTechBNT162b2	203	No	Yes	N/A	N/A
Control	Female	60	Pfizer BioNTechBNT162b2	229	Yes, 113 days after COVID-19, WHO score 2	Yes	N/A	N/A

Abbreviations: CVID—common variable immunodeficiency; SIgAD—selective IgA deficiency; SARS-CoV-2—severe acute respiratory syndrome coronavirus 2; COVID-19—coronavirus disease 2019; WHO—World Health Organization. * Ambulatory mild disease: score 1—asymptomatic, viral ribonucleic acid (RNA) detected; score 2—symptomatic, independent; score 3—symptomatic, assistance needed; Hospitalized: moderate disease: score 4—hospitalized, no oxygen therapy required; score 5—hospitalized, oxygen by mask or nasal prongs; Hospitalized: severe disease: score 6—hospitalized with non-invasive ventilation or high flow oxygen; score 7—intubation and mechanic ventilation partial pressure of oxygen/fraction of inspired oxygen ratio (pO2/FiO2) ≥ 150 or oxygen saturation/fraction of inspired oxygen ratio (SpO2/FiO2) ≥ 200; score 8—mechanic ventilation pO2/FiO2 < 150 or SpO2/ FiO2 < 200 or vasopressors; score 9—mechanic ventilation pO2/FiO2 < 150 or SpO2/FiO2 < 200 and vasopressors, dialysis, or extracorporeal membrane oxygenation (ECMO); Dead: score 10—dead.

## Data Availability

The datasets generated during and/or analyzed during the current study are available from the corresponding author upon reasonable request.

## Supplementary Materials

The following supporting information can be downloaded at: https://www.mdpi.com/article/10.3390/v15051146/s1. Table S1. Changes in cytokine levels following SARS-CoV-2 stimulation. Table S2. Predictors of COVID-19 during the follow-up period in PAD patients. Table S3. Associations between the cytokine levels and previous SARS-CoV-2 vaccination. Table S4. Associations between the changes in cytokine levels following SARS-CoV-2 antigen stimulation and SARS-CoV-2-specific humoral response. Table S5. Associations between the cytokine levels and patient’s demographic and clinical characteristics. Table S6. Spearman correlation analysis.

## References

[1] Tangye S.G., Al-Herz W., Bousfiha A., Cunningham-Rundles C., Franco J.L., Holland S.M., Klein C., Morio T., Oksenhendler E., Picard C. (2022). Human Inborn Errors of Immunity: 2022 Update on the Classification from the International Union of Immunological Societies Expert Committee. J. Clin. Immunol..

[2] Yazdani R., Azizi G., Abolhassani H., Aghamohammadi A. (2017). Selective IgA Deficiency: Epidemiology, Pathogenesis, Clinical Phenotype, Diagnosis, Prognosis and Management. Scand. J. Immunol..

[3] Seidel M.G., Kindle G., Gathmann B., Quinti I., Buckland M., van Montfrans J., Scheible R., Rusch S., Gasteiger L.M., Grimbacher B. (2019). The European Society for Immunodeficiencies (ESID) Registry Working Definitions for the Clinical Diagnosis of Inborn Errors of Immunity. J. Allergy Clin. Immunol. Pract..

[4] Ho H.-E., Cunningham-Rundles C. (2020). Non-infectious Complications of Common Variable Immunodeficiency: Updated Clinical Spectrum, Sequelae, and Insights to Pathogenesis. Front. Immunol..

[5] Galant-Swafford J. (2021). Selective Immunoglobulin A Deficiency and the Microbiome. Crit. Rev. Immunol..

[6] Fernando S.L., Jang H.S.-I., Li J. (2021). The Immune Dysregulation of Common Variable Immunodeficiency Disorders. Immunol. Lett..

[7] Varzaneh F.N., Keller B., Unger S., Aghamohammadi A., Warnatz K., Rezaei N. (2014). Cytokines in Common Variable Immunodeficiency as Signs of Immune Dysregulation and Potential Therapeutic Targets—A Review of the Current Knowledge. J. Clin. Immunol..

[8] Zhou P., Yang X.-L., Wang X.-G., Hu B., Zhang L., Zhang W., Si H.-R., Zhu Y., Li B., Huang C.-L. (2020). A pneumonia outbreak associated with a new coronavirus of probable bat origin. Nature.

[9] Gupta K., Gandhi S., Mebane A., Singh A., Vishnuvardhan N., Patel E. (2021). Cancer patients and COVID-19: Mortality, serious complications, biomarkers, and ways forward. Cancer Treat. Res. Commun..

[10] Raja M.A., Mendoza M.A., Villavicencio A., Anjan S., Reynolds J.M., Kittipibul V., Fernandez A., Guerra G., Camargo J.F., Simkins J. (2020). COVID-19 in solid organ transplant recipients: A systematic review and meta-analysis of current literature. Transplant. Rev..

[11] Hultcrantz M., Richter J., Rosenbaum C.A., Patel D., Smith E.L., Korde N., Lu S.X., Mailankody S., Shah U.A., Lesokhin A.M. (2020). COVID-19 Infections and Clinical Outcomes in Patients with Multiple Myeloma in New York City: A Cohort Study from Five Academic Centers. Blood Cancer Discov..

[12] Malle L., Gao C., Hur C., Truong H.Q., Bouvier N.M., Percha B., Kong X.-F., Bogunovic D. (2020). Individuals with Down syndrome hospitalized with COVID-19 have more severe disease. Anesth. Analg..

[13] Brown L.B., Spinelli M.A., Gandhi M. (2021). The interplay between HIV and COVID-19: Summary of the data and responses to date. Curr. Opin. HIV AIDS.

[14] Shields A.M., Burns S.O., Savic S., Richter A.G., Anantharachagan A., Arumugakani G., Baker K., Bahal S., Bermingham W., Bhole M. (2021). COVID-19 in patients with primary and secondary immunodeficiency: The United Kingdom experience. J. Allergy Clin. Immunol..

[15] Bucciol G., Tangye S.G., Meyts I. (2021). Coronavirus disease 2019 in patients with inborn errors of immunity: Lessons learned. Curr. Opin. Pediatr..

[16] Delavari S., Abolhassani H., Abolnezhadian F., Babaha F., Iranparast S., Ahanchian H., Moazzen N., Nabavi M., Arshi S., Fallahpour M. (2021). Impact of SARS-CoV-2 Pandemic on Patients with Primary Immunodeficiency. J. Clin. Immunol..

[17] Schulz E., Hodl I., Forstner P., Hatzl S., Sareban N., Moritz M., Fessler J., Dreo B., Uhl B., Url C. (2021). CD19+IgD+CD27- Naïve B Cells as Predictors of Humoral Response to COVID 19 mRNA Vaccination in Immunocompromised Patients. Front. Immunol..

[18] Hagin D., Freund T., Navon M., Halperin T., Adir D., Marom R., Levi I., Benor S., Alcalay Y., Freund N.T. (2021). Immunogenicity of Pfizer-BioNTech COVID-19 vaccine in patients with inborn errors of immunity. J. Allergy Clin. Immunol..

[19] Amodio D., Ruggiero A., Sgrulletti M., Pighi C., Cotugno N., Medri C., Morrocchi E., Colagrossi L., Russo C., Zaffina S. (2021). Humoral and Cellular Response Following Vaccination with the BNT162b2 mRNA COVID-19 Vaccine in Patients Affected by Primary Immunodeficiencies. Front. Immunol..

[20] Salinas A.F., Mortari E.P., Terreri S., Quintarelli C., Pulvirenti F., Di Cecca S., Guercio M., Milito C., Bonanni L., Auria S. (2021). SARS-CoV-2 Vaccine Induced Atypical Immune Responses in Antibody Defects: Everybody Does their Best. J. Clin. Immunol..

[21] Shields A.M., Faustini S.E., Hill H.J., Al-Taei S., Tanner C., Ashford F., Workman S., Moreira F., Verma N., Wagg H. (2022). SARS-CoV-2 Vaccine Responses in Individuals with Antibody Deficiency: Findings from the COV-AD Study. J. Clin. Immunol..

[22] Bergman P., Blennow O., Hansson L., Mielke S., Nowak P., Chen P., Söderdahl G., Österborg A., Smith C.I.E., Wullimann D. (2021). Safety and efficacy of the mRNA BNT162b2 vaccine against SARS-CoV-2 in five groups of immunocompromised patients and healthy controls in a prospective open-label clinical trial. Ebiomedicine.

[23] Squire J., Joshi A. (2021). Seroconversion after coronavirus disease 2019 vaccination in patients with immune deficiency. Ann. Allergy Asthma Immunol..

[24] Pham M.N., Murugesan K., Banaei N., Pinsky B.A., Tang M., Hoyte E., Lewis D.B., Gernez Y. (2022). Immunogenicity and tolerability of COVID-19 messenger RNA vaccines in primary immunodeficiency patients with functional B-cell defects. J. Allergy Clin. Immunol..

[25] Lucane Z., Slisere B., Ozola L., Rots D., Papirte S., Vilne B., Gailite L., Kurjane N. (2023). Long-Term Immunological Memory of SARS-CoV-2 Is Present in Patients with Primary Antibody Deficiencies for up to a Year after Vaccination. Vaccines.

[26] van Leeuwen L.P.M., GeurtsvanKessel C.H., Ellerbroek P.M., de Bree G.J., Potjewijd J., Rutgers A., Jolink H., van de Veerdonk F., van Gorp E.C.M., de Wilt F. (2022). Immunogenicity of the mRNA-1273 COVID-19 vaccine in adult patients with inborn errors of immunity. J. Allergy Clin. Immunol..

[27] Leung D., Mu X., Duque J.S.R., Cheng S.M.S., Wang M., Zhang W., Zhang Y., Tam I.Y.S., Lee T.S.S., Lam J.H.Y. (2022). Safety and immunogenicity of 3 doses of BNT162b2 and CoronaVac in children and adults with inborn errors of immunity. Front. Immunol..

[28] Erra L., Uriarte I., Colado A., Paolini M.V., Seminario G., Fernández J.B., Tau L., Bernatowiez J., Moreira I., Vishnopolska S. (2022). COVID-19 Vaccination Responses with Different Vaccine Platforms in Patients with Inborn Errors of Immunity. J. Clin. Immunol..

[29] Abo-Helo N., Muhammad E., Ghaben-Amara S., Panasoff J., Cohen S. (2021). Specific antibody response of patients with common variable immunodeficiency to BNT162b2 coronavirus disease 2019 vaccination. Ann. Allergy Asthma Immunol..

[30] Arroyo-Sánchez D., Cabrera-Marante O., Laguna-Goya R., Almendro-Vázquez P., Carretero O., Gil-Etayo F.J., Suàrez-Fernández P., Pérez-Romero P., Rodríguez de Frías E., Serrano A. (2022). Immunogenicity of Anti-SARS-CoV-2 Vac-cines in Common Variable Immunodeficiency. J. Clin. Immunol..

[31] Ponsford M.J., Evans K., Carne E.M., Jolles S., Bramhall K., Grant L., McGuire F., Matthews A., Bradley R., Wijetilleka S. (2022). COVID-19 Vaccine Uptake and Efficacy in a National Immunodeficiency Cohort. J. Clin. Immunol..

[32] Antolí A., Rocamora-Blanch G., Framil M., Mas-Bosch V., Navarro S., Bermudez C., Martinez-Yelamos S., Dopico E., Calatayud L., Garcia-Muñoz N. (2022). Evaluation of Humoral and Cellular Immune Responses to the SARS-CoV-2 Vaccine in Patients with Common Variable Immunodeficiency Phenotype and Patient Receiving B-Cell Depletion Therapy. Front. Immunol..

[33] Zhang J.-Y., Wang X.-M., Xing X., Xu Z., Zhang C., Song J.-W., Fan X., Xia P., Fu J.-L., Wang S.-Y. (2020). Single-cell landscape of immunological responses in patients with COVID-19. Nat. Immunol..

[34] Aiello A., Coppola A., Vanini V., Petrone L., Cuzzi G., Salmi A., Altera A.M.G., Tortorella C., Gualano G., Gasperini C. (2022). Accuracy of QuantiFERON SARS-CoV-2 research use only assay and characterization of the CD4+ and CD8+ T cell-SARS-CoV-2 response: Comparison with a homemade interferon-γ release assay. Int. J. Infect. Dis..

[35] Pegu A., O’connell S.E., Schmidt S.D., O’dell S., Talana C.A., Lai L., Albert J., Anderson E., Bennett H., Corbett K.S. (2021). Durability of mRNA-1273 vaccine–induced antibodies against SARS-CoV-2 variants. Science.

[36] Moss P. (2022). The T cell immune response against SARS-CoV-2. Nat. Immunol..

[37] Kunnumakkara A.B., Rana V., Parama D., Banik K., Girisa S., Henamayee S., Thakur K.K., Dutta U., Garodia P., Gupta S.C. (2021). COVID-19, cytokines, inflammation, and spices: How are they related?. Life Sci..

[38] Tang L., Yin Z., Hu Y., Mei H. (2020). Controlling Cytokine Storm Is Vital in COVID-19. Front. Immunol..

[39] Queiroz M.A.F., das Neves P.F.M., Lima S.S., Lopes J.D.C., Torres M.K.D.S., Vallinoto I.M.V.C., Bichara C.D.A., dos Santos E.F., de Brito M.T.F.M., da Silva A.L.S. (2022). Cytokine Profiles Associated with Acute COVID-19 and Long COVID-19 Syndrome. Front. Cell. Infect. Microbiol..

[40] Huang C., Wang Y., Li X., Ren L., Zhao J., Hu Y., Zhang L., Fan G., Xu J., Gu X. (2020). Clinical features of patients infected with 2019 novel coronavirus in Wuhan, China. Lancet.

[41] Chen G., Wu D., Guo W., Cao Y., Huang D., Wang H., Wang T., Zhang X., Chen H., Yu H. (2020). Clinical and immunological features of severe and moderate coronavirus disease 2019. J. Clin. Investig..

[42] Qin C., Zhou L., Hu Z., Zhang S., Yang S., Tao Y., Xie C., Ma K., Shang K., Wang W. (2020). Dysregulation of Immune Response in Patients with Coronavirus 2019 (COVID-19) in Wuhan, China. Clin. Infect. Dis..

[43] Olajide O.A., Iwuanyanwu V.U., Lepiarz-Raba I., Al-Hindawi A.A. (2021). Induction of Exaggerated Cytokine Production in Human Peripheral Blood Mononuclear Cells by a Recombinant SARS-CoV-2 Spike Glycoprotein S1 and Its Inhibition by Dexamethasone. Inflammation.

[44] Diao B., Wang C., Tan Y., Chen X., Liu Y., Ning L., Chen L., Li M., Liu Y., Wang G. (2020). Reduction and Functional Exhaustion of T Cells in Patients with Coronavirus Disease 2019 (COVID-19). Front. Immunol..

[45] Petruccioli E., Fard S.N., Navarra A., Petrone L., Vanini V., Cuzzi G., Gualano G., Pierelli L., Bertoletti A., Nicastri E. (2021). Exploratory analysis to identify the best antigen and the best immune biomarkers to study SARS-CoV-2 infection. J. Transl. Med..

[46] Conti P., Ronconi G., Caraffa A., Gallenga C.E., Ross R., Frydas I., Kritas S.K. (2020). Induction of pro-inflammatory cytokines (IL-1 and IL-6) and lung inflammation by Coronavirus-19 (CoV-19 or SARS-CoV-2): Anti-inflammatory strategies. J. Biol. Regul. Homeost. Agents.

[47] Hu H., Pan H., Li R., He K., Zhang H., Liu L. (2022). Increased Circulating Cytokines Have a Role in COVID-19 Severity and Death with a More Pronounced Effect in Males: A Systematic Review and Meta-Analysis. Front. Pharmacol..

[48] Perreau M., Suffiotti M., Marques-Vidal P., Wiedemann A., Levy Y., Laouénan C., Ghosn J., Fenwick C., Comte D., Roger T. (2021). The cytokines HGF and CXCL13 predict the severity and the mortality in COVID-19 patients. Nat. Commun..

[49] Declercq J., De Leeuw E., Lambrecht B.N. (2022). Inflammasomes and IL-1 family cytokines in SARS-CoV-2 infection: From prognostic marker to therapeutic agent. Cytokine.

[50] Dhar S.K., Vishnupriyan K., Damodar S., Gujar S., Das M. (2021). IL-6 and IL-10 as predictors of disease severity in COVID-19 patients: Results from meta-analysis and regression. Heliyon.

[51] Cruz A.S., Mendes-Frias A., Oliveira A.I., Dias L., Matos A.R., Carvalho A., Capela C., Pedrosa J., Gil Castro A., Silvestre R. (2021). Interleukin-6 Is a Biomarker for the Development of Fatal Severe Acute Respiratory Syndrome Coronavirus 2 Pneumonia. Front. Immunol..

[52] Zucker J., Gomez-Simmonds A., Purpura L.J., Shoucri S., LaSota E., Morley N.E., Sovic B.W., Castellon M.A., Theodore D.A., Bartram L.L. (2021). Supervised Machine Learning Approach to Identify Early Predictors of Poor Outcome in Patients with COVID-19 Presenting to a Large Quaternary Care Hospital in New York City. J. Clin. Med..

[53] Tveita A., Murphy S.L., Holter J.C., Kildal A.B., Michelsen A.E., Lerum T.V., Kaarbø M., Heggelund L., Holten A.R., Finbråten A.K. (2022). High Circulating Levels of the Homeostatic Chemokines CCL19 and CCL21 Predict Mortality and Disease Severity in COVID-19. J. Infect. Dis..

[54] Gatselis N.K., Lygoura V., Lyberopoulou A., Giannoulis G., Samakidou A., Vaiou A., Vatidis G., Antoniou K., Stefos A., Georgiadou S. (2022). Soluble IL-2R Levels at Baseline Predict the Development of Severe Respiratory Failure and Mortality in COVID-19 Patients. Viruses.

[55] Prada L.S.-D., Gorgojo-Galindo Ó., Fierro I., Martínez-García A.M., de Quintana G.S.-L., Gutiérrez-Bustillo R., Pelaez-Jareño M.T., Álvarez-Fuente E., Gómez-Sánchez E., Tamayo E. (2022). Time evolution of cytokine profiles associated with mortality in COVID-19 hospitalized patients. Front. Immunol..

[56] Velavan T.P., Kuk S., Linh L.T.K., Calle C.L., Lalremruata A., Pallerla S.R., Kreidenweiss A., Held J., Esen M., Gabor J. (2021). Longitudinal monitoring of laboratory markers characterizes hospitalized and ambulatory COVID-19 patients. Sci. Rep..

[57] Satış H., Özger H.S., Yıldız P.A., Hızel K., Gulbahar Ö., Erbaş G., Aygencel G., Tunccan O.G., Öztürk M.A., Dizbay M. (2021). Prognostic value of interleukin-18 and its association with other inflammatory markers and disease severity in COVID-19. Cytokine.

[58] Xiao N., Nie M., Pang H., Wang B., Hu J., Meng X., Li K., Ran X., Long Q., Deng H. (2021). Integrated cytokine and metabolite analysis reveals immunometabolic reprogramming in COVID-19 patients with therapeutic implications. Nat. Commun..

[59] Mohamed H.A., Abdelkafy A.E., Khairy R.M.M., Abdelraheim S.R., Kamel B.A., Marey H. (2023). MicroRNAs and cytokines as potential predictive biomarkers for COVID-19 disease progression. Sci. Rep..

[60] Babaha F., Rezaei N. (2020). Primary Immunodeficiency Diseases in COVID-19 Pandemic: A Predisposing or Protective Factor?. Am. J. Med. Sci..

[61] Jalil M., Pietras J., Ahmed S.N., Daniels P., Hostoffer R. (2022). COVID-19 Infection in Patients with Humoral Immunodeficiency: A Case Series and Literature Review. Allergy Rhinol..

[62] Odnoletkova I., Kindle G., Quinti I., Grimbacher B., Knerr V., Gathmann B., Ehl S., Mahlaoui N., Van Wilder P., Bogaerts K. (2018). The burden of common variable immunodeficiency disorders: A retrospective analysis of the European Society for Immunodeficiency (ESID) registry data. Orphanet J. Rare Dis..

[63] Martinson N., Gordhan B., Petkov S., Pillay A.-D., Seiphetlo T., Singh N., Otwombe K., Lebina L., Fredolini C., Chiodi F. (2023). Proteomic Analysis of Mucosal and Systemic Responses to SARS-CoV-2 Antigen. Vaccines.

[64] Hu Z., van der Ploeg K., Chakraborty S., Arunachalam P.S., Mori D.A., Jacobson K.B., Bonilla H., Parsonnet J., Andrews J.R., Holubar M. (2022). Early immune markers of clinical, virological, and immunological outcomes in patients with COVID-19: A multi-omics study. Elife.

[65] Chia W.N., Zhu F., Ong S.W.X., Young B.E., Fong S.-W., Le Bert N., Tan C.W., Tiu C., Zhang J., Tan S.Y. (2021). Dynamics of SARS-CoV-2 neutralising antibody responses and duration of immunity: A longitudinal study. Lancet Microbe.

[66] Marshall J.C., Murthy S., Diaz J., Adhikari N.K., Angus D.C., Arabi Y.M., Baillie K., Bauer M., Berry S., Blackwood B. (2020). A minimal common outcome measure set for COVID-19 clinical research. Lancet Infect. Dis..

[67] Wehr C., Kivioja T., Schmitt C., Ferry B., Witte T., Eren E., Vlkova M., Hernandez-Gonzalez M., Detkova D., Bos P.R. (2008). The EUROclass trial: Defining subgroups in common variable immunodeficiency. Blood.

[68] Ameratunga R. (2018). Assessing Disease Severity in Common Variable Immunodeficiency Disorders (CVID) and CVID-Like Disorders. Front. Immunol..

[69] Tormo N., Navalpotro D., Martínez-Serrano M., Moreno M., Grosson F., Tur I., Guna M.R., Soriano P., Tornero A., Gimeno C. (2022). Commercial Interferon-gamma release assay to assess the immune response to first and second doses of mRNA vaccine in previously COVID-19 infected versus uninfected individuals. Diagn. Microbiol. Infect. Dis..

[70] Pekayvaz K., Leunig A., Kaiser R., Joppich M., Brambs S., Janjic A., Popp O., Nixdorf D., Fumagalli V., Schmidt N. (2022). Protective immune trajectories in early viral containment of non-pneumonic SARS-CoV-2 infection. Nat. Commun..

[71] Dijssel J.V.D., Hagen R.R., de Jongh R., Steenhuis M., Rispens T., Geerdes D.M., Mok J.Y., Kragten A.H., Duurland M.C., Verstegen N.J. (2022). Parallel detection of SARS-CoV-2 epitopes reveals dynamic immunodominance profiles of CD8 ^+^ T memory cells in convalescent COVID -19 donors. Clin. Transl. Immunol..

[72] Jørgensen S.F., Fevang B., Aukrust P. (2019). Autoimmunity and Inflammation in CVID: A Possible Crosstalk between Immune Activation, Gut Microbiota, and Epigenetic Modifications. J. Clin. Immunol..

[73] Bergamaschi C., Terpos E., Rosati M., Angel M., Bear J., Stellas D., Karaliota S., Apostolakou F., Bagratuni T., Patseas D. (2021). Systemic IL-15, IFN-γ and IP-10/CXCL10 Signature Associated with Effective Immune Response to SARS-CoV-2 in BNT162b2 mRNA Vaccine Recipients. Cell Rep..

[74] Li Z., Xiang T., Liang B., Deng H., Wang H., Feng X., Quan X., Wang X., Li S., Lu S. (2021). Characterization of SARS-CoV-2-Specific Humoral and Cellular Immune Responses Induced by Inactivated COVID-19 Vaccines in a Real-World Setting. Front. Immunol..

[75] Flego D., Cesaroni S., Romiti G.F., Corica B., Marrapodi R., Scafa N., Maiorca F., Lombardi L., Pallucci D., Pulcinelli F. (2022). Platelet and immune signature associated with a rapid response to the BNT162b2 mRNA COVID-19 vaccine. J. Thromb. Haemost..

[76] Tahtinen S., Tong A.-J., Himmels P., Oh J., Paler-Martinez A., Kim L., Wichner S., Oei Y., McCarron M.J., Freund E.C. (2022). IL-1 and IL-1ra are key regulators of the inflammatory response to RNA vaccines. Nat. Immunol..

[77] Schultheiß C., Willscher E., Paschold L., Gottschick C., Klee B., Henkes S.-S., Bosurgi L., Dutzmann J., Sedding D., Frese T. (2022). The IL-1β, IL-6, and TNF cytokine triad is associated with post-acute sequelae of COVID-19. Cell Rep. Med..

[78] Sahin U., Muik A., Vogler I., Derhovanessian E., Kranz L.M., Vormehr M., Quandt J., Bidmon N., Ulges A., Baum A. (2021). BNT162b2 vaccine induces neutralizing antibodies and poly-specific T cells in humans. Nature.

[79] Ramírez-Martínez G., Jiménez-Álvarez L.A., Cruz-Lagunas A., Ignacio-Cortés S., Gómez-García I.A., Rodríguez-Reyna T.S., Choreño-Parra J.A., Zúñiga J. (2022). Possible Role of Matrix Metalloproteinases and TGF-β in COVID-19 Severity and Sequelae. J. Interf. Cytokine Res..

[80] Xu A., Liu Y., Chen W., Wang J., Xue Y., Huang F., Rong L., Lin J., Liu D., Yan M. (2016). TGF-β–Induced Regulatory T Cells Directly Suppress B Cell Responses through a Noncytotoxic Mechanism. J. Immunol..

[81] Strainic M.G., Shevach E.M., An F., Lin F., Medof M.E. (2013). Absence of signaling into CD4+ cells via C3aR and C5aR enables autoinductive TGF-β1 signaling and induction of Foxp3+ regulatory T cells. Nat. Immunol..

[82] Ferreira-Gomes M., Kruglov A., Durek P., Heinrich F., Tizian C., Heinz G.A., Pascual-Reguant A., Du W., Mothes R., Fan C. (2021). SARS-CoV-2 in severe COVID-19 induces a TGF-β-dominated chronic immune response that does not target itself. Nat. Commun..

[83] Villalba M.C.M., Ramírez O.V., Jiménez M.M., Garcia A.A., Alfonso J.M., Baéz G.G., Arrieta R.R., Simón D.R., Gainza D.A., Vázquez B.S. (2020). Interferon gamma, TGF-β1 and RANTES expression in upper airway samples from SARS-CoV-2 infected patients. Clin. Immunol..

[84] Hamidi S.H., Veethil S.K., Hamidi S.H. (2021). Role of pirfenidone in TGF-β pathways and other inflammatory pathways in acute respiratory syndrome coronavirus 2 (SARS-CoV-2) infection: A theoretical perspective. Pharmacol. Rep..

[85] Yan T., Tan Y., Deng G., Sun Z., Liu B., Wang Y., Yuan F., Sun Q., Hu P., Gao L. (2022). TGF-β induces GBM mesenchymal transition through upregulation of CLDN4 and nuclear translocation to activate TNF-α/NF-κB signal pathway. Cell Death Dis..

[86] Petrone L., Petruccioli E., Vanini V., Cuzzi G., Fard S.N., Alonzi T., Castilletti C., Palmieri F., Gualano G., Vittozzi P. (2021). A whole blood test to measure SARS-CoV-2-specific response in COVID-19 patients. Clin. Microbiol. Infect..

[87] Tan A.T., Lim J.M., Le Bert N., Kunasegaran K., Chia A., Qui M.D., Tan N., Ni Chia W., de Alwis R., Ying D. (2021). Rapid measurement of SARS-CoV-2 spike T cells in whole blood from vaccinated and naturally infected individuals. J. Clin. Investig..

[88] Schultheiß C., Paschold L., Willscher E., Simnica D., Wöstemeier A., Muscate F., Wass M., Eisenmann S., Dutzmann J., Keyßer G. (2021). Maturation trajectories and transcriptional landscape of plasmablasts and autoreactive B cells in COVID-19. iScience.

[89] Lyons-Weiler J. (2020). Pathogenic priming likely contributes to serious and critical illness and mortality in COVID-19 via autoimmunity. J. Transl. Autoimmun..

[90] Shoenfeld Y. (2020). Corona (COVID-19) time musings: Our involvement in COVID-19 pathogenesis, diagnosis, treatment and vaccine planning. Autoimmun. Rev..

[91] Kasperkiewicz M., Woodley D.T. (2023). COVID-19 and autoimmune bullous diseases: Lessons learned. Autoimmun. Rev..

[92] Laxminarayana D. (2022). Molecular insights into onset of autoimmunity in SARS-CoV-2 infected patients. Rheumatol. Autoimmun..

[93] Catriona C., Paolo P. (2022). SARS-CoV-2 induced post-translational protein modifications: A trigger for developing autoimmune diabetes?. Diabetes/Metab. Res. Rev..

[94] Halpert G., Shoenfeld Y. (2020). SARS-CoV-2, the autoimmune virus. Autoimmun. Rev..

[95] Zhang Y., Xiao M., Zhang S., Xia P., Cao W., Jiang W., Chen H., Ding X., Zhao H., Zhang H. (2020). Coagulopathy and Antiphospholipid Antibodies in Patients with COVID-19. N. Engl. J. Med..

[96] Ueno H. (2016). Human Circulating T Follicular Helper Cell Subsets in Health and Disease. J. Clin. Immunol..

[97] Yazdani R., Abolhassani H., Kiaee F., Habibi S., Azizi G., Tavakol M., Chavoshzadeh Z., Mahdaviani S.A., Momen T., Gharagozlou M. (2019). Comparison of Common Monogenic Defects in a Large Predominantly Antibody Deficiency Cohort. J. Allergy Clin. Immunol. Pract..

[98] Singh K., Chang C., Gershwin M.E. (2014). IgA deficiency and autoimmunity. Autoimmun. Rev..

[99] Salzer E., Kansu A., Sic H., Májek P., Ikincioğullari A., Dogu F.E., Prengemann N.K., Santos-Valente E., Pickl W.F., Bilic I. (2014). Early-onset inflammatory bowel disease and common variable immunodeficiency–like disease caused by IL-21 deficiency. J. Allergy Clin. Immunol..

[100] Desjardins M., Béland M., Dembele M., Lejtenyi D., Drolet J.-P., Lemire M., Tsoukas C., Ben-Shoshan M., Noya F.J.D., Alizadehfar R. (2017). Modulation of the Interleukin-21 Pathway with Interleukin-4 Distinguishes Common Variable Immunodeficiency Patients with More Non-infectious Clinical Complications. J. Clin. Immunol..

[101] Coraglia A., Galassi N., Fernández Romero D.S., Juri M.C., Felippo M., Malbran A., De Bracco M.M.E. (2016). Common Variable Immunodeficiency and Circulating TFH. J. Immunol. Res..

[102] Warnatz K., Bossaller L., Salzer U., Skrabl-Baumgartner A., Schwinger W., van der Burg M., van Dongen J.J.M., Orlowska-Volk M., Knoth R., Durandy A. (2006). Human ICOS deficiency abrogates the germinal center reaction and provides a monogenic model for common variable immunodeficiency. Blood.

[103] de Lollo C., Vasconcelos D.d.M., da Silva Oliveira L.M., de Oliveira Titz T., Carneiro-Sampaio M., Jacob C.M.A., da Silva Duarte A.J., Sato M.N. (2016). Impaired CD8+ T Cell Responses upon Toll-like Receptor Activation in Common Variable Immuno-deficiency. J. Transl. Med..

